# The under‐appreciated world of the serpin family of serine proteinase inhibitors

**DOI:** 10.15252/emmm.202217144

**Published:** 2023-05-09

**Authors:** Marie‐Christine Bouton, Margarethe Geiger, William P Sheffield, James A Irving, David A Lomas, Sihong Song, Ritvik S Satyanarayanan, Liqiang Zhang, Grant McFadden, Alexandra R Lucas

**Affiliations:** ^1^ Université Paris Cité and Université Sorbonne Paris Nord, INSERM U1148‐LVTS Paris France; ^2^ Center for Physiology and Pharmacology, Department of Vascular Biology and Thrombosis Research Medical University Vienna Vienna Austria; ^3^ McMaster University and Canadian Blood Services Hamilton ON Canada; ^4^ UCLRespiratory, Division of Medicine University College London London UK; ^5^ Department of Pharmaceutics, College of Pharmacy University of Florida Gainesville FL USA; ^6^ Biomedical Engineering Arizona State University (ASU) Tempe AZ USA; ^7^ Center for Personalized Diagnostics (CPD), Biodesign Institute Arizona State University (ASU) Tempe AZ USA; ^8^ Translational Drug Development (TD2) Inc Scottsdale AZ USA; ^9^ Center for Immunotherapy, Vaccines and Virotherapy (CIVV), Biodesign Institute Arizona State University (ASU) Tempe AZ USA

**Keywords:** Microbiology, Virology & Host Pathogen Interaction, Pharmacology & Drug Discovery

## Abstract

In the practice of medicine, many fundamental biological pathways that require tight on/off control, such as inflammation and circulatory homeostasis, are regulated by serine proteinases, but we rarely consider the unique protease inhibitors that, in turn, regulate these proteases. The serpins are a family of proteins with a shared tertiary structure, whose members largely act as serine protease inhibitors, found in all forms of life, ranging from viruses, bacteria, and archaea to plants and animals. These proteins represent up to 2–10% of proteins in the human blood and are the third most common protein family.

## Graphical abstract

In this commentary, A. Lucas and colleagues discuss the biological functions of serine protease inhibitors (serpins) and their therapeutic potential for the treatment of various diseases.

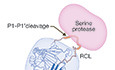

The term “serpin,” first coined by Robin Carrell and Jim Travis in 1985, reflects the common evolutionary ancestry and by extension a shared tertiary structure and mechanism between family members that are classified into 16 subfamilies (A to P) according to inferred phylogenetic relationships (Silverman *et al*, 2001). Despite their name, some serpins have diverged to become transporters of proteins, small molecules such as hormones, or to target other classes of protease. Deficiency or dysfunction of serpins, as in some genetic disorders, cause severe clinical disorders: antithrombin III (AT, SERPINC1), alpha‐1‐antitrypsin (A1AT, SERPINA1), complement C1 esterase‐inhibitor (C1INH, SERPING1), and neuroserpin (NSP, SERPINI1) are associated with severe clotting disorders, severe emphysema, angioedema and dementia, epilepsy, and neurodegeneration, respectively.

What is often not discussed are the unique properties of serpins that allow these proteins to function so efficiently as powerful and widely effective regulators of central cardiovascular, hematological and immune response pathways—balancing thrombosis (clot formation) and thrombolysis (clot dissolution) in hemostasis, vascular response, lung function, neuronal signaling, and inflammation, among many functions. The prevalence of serpins in diverse physiological and evolutionary contexts corresponds with four central characteristics: (i) their role as inhibitors, acting specifically at sites where there is increased target protease activity; (ii) a unique “suicide” inhibitory mechanism of action; (iii) the ability to target a diverse array of protease targets; (iv) the amenability of the serpin structure to allosteric fine‐tuning of inhibitory activity (Maas & de Maat, 2021).

This inhibitory mechanism has been extensively investigated. Serpins that function as protease inhibitors are folded into a high‐energy structure that, upon interaction with a target protease, expends this energy by incorporating the cleaved reactive center loop (RCL) into their central A β‐sheet. The result is a covalent inhibitory serpin‐protease complex, in which the activity of both is lost, termed suicide inhibition. Some serpins, such as the thrombolysis regulator plasminogen activator inhibitor‐1 (PAI‐1, SERPINE1), can also exist in a third state where fluidity of the RCL allows insertion into the A β‐sheet in the absence of cleavage, resulting in a latent, inactive low‐energy state (Maas & de Maat, 2021; Silverman *et al*, 2001). The process of RCL presentation or insertion is, in some cases, modulated by a ligand, such as heparin in the case of antithrombin, and vitronectin with PAI‐1. Some serpins have a primary protease target but, for many, the regulated proteases are often diverse and numerous, as for PAI‐1 and A1AT and with the virus‐derived serpins. Most serpins function in the presence of active proteases and thus operate exclusively at sites where there is excessive target protease activity. It is this spatially defined regulation of central pathways that maintains normal hemostasis and makes serpins candidates as attractive therapeutic targets, or as therapeutics in their own right.

There are a wide variety of genetic mutations in serpins that have significant pathological sequelae. One of the more fascinating observations has been made by Lomas and colleagues and describes a mechanism through which genetic mutations in serpins can cause loss of inhibitory function. The RCL of genetically aberrant serpins can insert into an adjacent serpin β sheet forming inactive protein aggregates (or polymers), a repeating intermolecular domain swap such that the usual target protease pathways no longer function (Lomas & Carrell, 2002). These aggregates are both inactive and largely are retained by the producing cell, causing a corresponding deficit in inhibitor availability and deficiency in function. Such a process has been observed where C1Inh is genetically modified causing angioedema and in the genetic aberrancy of NSP where the accumulation of cerebral polymers causes dementia, epilepsy, and neurodegeneration. The main physiological role for A1AT is protection of the extracellular matrix of the lung from proteolytic damage arising from the release of proteases during an inflammatory response, primarily neutrophil elastase but with some reactivity against proteinase 3 and cathepsin G. When A1AT forms aggregates of inactive polymers, these molecules deposit within the liver, causing cirrhosis, and the lack of circulating A1AT leads to proteolytic dysregulation within the lung, predisposing to the development of emphysema. This has spurred the development of therapeutic strategies, including siRNA silencing and small molecule chaperones (Aymonnier *et al*, 2021; Lomas *et al*, 2021). By preventing the expression of A1AT, this suppresses polymer formation and reduces inclusions, but larger studies are required to demonstrate an effect on the progression of hepatic fibrosis. This also highlights the challenge of treating a condition that exhibits both gain‐of‐toxic function and loss‐of‐function phenotypes: By this strategy only, the former is addressed, and prevention of lung disease must be tackled by other means.

Due to variable presentation and progression of symptoms, some of which are shared with other respiratory conditions, A1AT deficiency is underdiagnosed, and when it is identified, the time between first symptoms and formal diagnosis can be several years. This condition is the leading genetic cause of liver failure requiring transplantation in infants and the main monogenic cause of chronic obstructive pulmonary disease. Weekly intravenous infusions of human plasma A1AT have been used to treat individuals with emphysema secondary to severe deficiency since 1987. This has a modest effect on the rate of progression of emphysema and is not licensed in some countries (including the UK) given concerns about cost‐effectiveness. The development of recombinant alternatives and possible delivery of therapy by inhalation or viral vectors are under investigation (Aymonnier *et al*, 2021; Maas & de Maat, 2021).

The capacity for serpins to be utilized as protein therapeutics is well illustrated by A1AT and also C1INH. A1AT gene therapies for lung and liver diseases have been developed and tested in clinical studies. The anti‐inflammatory and immune‐regulatory functions of A1AT have been studied both as a protein therapy and as adeno‐associated virus (AAV) gene therapy in autoimmune disease models (Song & Lu, 2018). These effects can also be observed in other inflammation‐associated models, including aging. A1AT has been studied both as a protein therapeutic and adeno‐associated virus (AAV) gene delivery in lung disease. Recently, A1AT has been investigated as a treatment for severe viral infections, specifically COVID‐19 (preprint: Zhang *et al*, 2022), and systemic lupus erythematosus‐associated lung disease and even aging. A1AT has also been shown to provide benefit in reducing heart damage in a preclinical model of myocardial infarction. These studies extend the potential utility of administered serpins beyond alleviation of a deficiency state as potential therapeutic agents in their own right. These serpins hold substantial therapeutic potential, particularly, variants engineered to target specific proteases. Serpin activity can be fine‐tuned by rational mutagenesis within the RCL and variants engineered to target specific proteases hold substantial therapeutic potential, illustrating the capacity for fine‐tuning of serpin activities. Introducing four additional mutations into A1AT M358R between P4 and P1' or between P3 and P3' redirected this serpin to inhibit only contact factor proteases (Maas & de Maat, 2021; Grover & Mackman, 2022) or only coagulation factor XIa (Hamada *et al*, 2021). The first variant improved outcomes in several murine models of thrombo‐inflammation, while the second has yet to be tested *in vivo*. Whether these modified serpins will make it to the clinic remains to be seen, but recombinant C1INH produced in the milk of transgenic rabbits is approved for the treatment of hereditary angioedema in some jurisdictions (Ruconest®, Salix Pharmaceuticals). AT produced in the milk of transgenic goats is FDA‐licensed for the treatment of AT‐deficient patients undergoing surgery or childbirth (ATryn, GTC Biotherapeutics).

In clinical medicine, a number of serpins are important endogenous regulators of hemostasis, including AT, heparin cofactor II, PAI‐1, protease nexin‐1 (PN‐1), and alpha 2 antiplasmin (A2AP, SERPINF2). The main circulating serpins regulating the balance between coagulation and fibrinolysis are, respectively, AT and PAI‐1. AT, the principal anticoagulant serpin, inhibits many coagulation proteases, including activated factors FIX, FVIII, FX, and thrombin. The inhibitory function of AT is tremendously accelerated by heparin, and its importance is highlighted by the fact that heparin and its derivatives are the most commonly used medications in clinical practice. Heparin is used to treat excessive clotting in cardiovascular disease, arising during events including myocardial infarction, deep venous thrombosis, and pulmonary embolism. AT has also been assessed as a therapeutic in bacterial sepsis (Aymonnier *et al*, 2021; Grover & Mackman, 2022), as has heparin activation of AT, but with borderline efficacy. PAI‐1 is a regulator of plasminogen activators, and above all is the principal antifibrinolytic serpin inhibiting tissue‐type plasminogen activator (tPA); tPA secreted by vascular endothelial cells is the major plasminogen activator playing a pivotal role in intravascular fibrinolysis. As well as being found in platelets, PAI‐1 is present free in blood, where the active form of PAI‐1 is stabilized by its binding to vitronectin, an abundant protein in plasma and within the extracellular matrix of the vessel wall (Grover & Mackman, 2022). In the presence of this cofactor or heparin, PAI‐1 has an expanded inhibitory function, binding and inhibiting thrombin. This serpin has roles in thrombo‐inflammation and is profibrotic in liver, lung, and kidney but is reported as protective in preventing hypertension‐induced cardiac fibrosis. PAI‐1 is a marker associated with increased cardiovascular disease, metabolic syndrome, coronary artery disease, and some cancers and for inflammatory disease states such as sepsis, severe COVID‐19 (SARS‐CoV2), and influenza infections. In a recent study, Reuland & Church (2020) reported that elevated PAI‐1 can reduce synuclein breakdown by proteases leading to increased protein aggregation and worsening of Parkinson's disease. Inhibition of PAI‐1 therefore seems to be a promising therapeutic tool in cardiovascular and other diseases, and several approaches have been made to develop drugs that could be used as PAI‐1 inhibitors. Inhibition of PAI‐1 activity can be achieved by preventing complex formation of PAI‐1 with target proteases, by altering the characteristics of PAI‐1 from an inhibitor of, to a substrate for, target proteases, or by inducing the inactive latent conformation of PAI‐1. Antibody‐based inhibitors, peptides, and other small molecules have been studied *in vitro* and investigated for their therapeutic potential in preclinical models (Sillen & Declerck, 2020). Although there are some ongoing clinical trials, none of the PAI‐1 inhibitors has been approved for therapeutical use in humans so far.

Besides PAI‐1, A2AP is another antifibrinolytic serpin central to homeostasis in mammals (Singh *et al*, 2020). A2AP is primarily synthesized by the liver and released into the blood. Congenital A2AP serpin deficiency (Miyasato disease) causes spontaneous rebleeding, and A2AP is also associated with an increased risk of cardiovascular disease potentially by inhibiting proteolysis of fibrin clots. A2AP may reduce tPA treatment efficacy following cerebrovascular accidents and is associated with deep vein thrombosis (DVT) and pulmonary embolism. A2AP is conversely associated with reduced metastases and lymphatic remodeling in cancer models and is associated with neuronal dendrite outgrowth in the brain. A2AP deficiency is associated with impaired motor function, abnormal cognitive function, anxiety and depression in mice as well as increased fibrillar plaque in Alzheimer's models of disease. A2AP binds to fibrin as well as being an ultrafast inhibitor of plasmin; the relative role of these activities is a matter of debate. A2AP is associated with microvascular thrombosis and MMP9 expression, and thus, it is considered to have key roles in controlling thrombosis as well as fibrinolysis, demonstrating regulation of both clotting and bleeding pathways, and illustrates the potential for serpins to selectively target areas with increased protease activity. Strategies to inhibit A2AP with antibody‐mediated therapy or peptides are under investigation, and Phase 2 trials of antibody to A2AP for the treatment of PE are in the planning stages. As noted, activity of some serpins is modified by heparin and by citrullination. Serpins are highly citrullinated in some inflammatory diseases like rheumatoid arthritis. Interestingly, citrullination abolished the ability of AT or PAI‐1 to inhibit their target protease and converts A2AP from an inhibitor into a substrate (Singh *et al*, 2020; Grover & Mackman, 2022). Thus, AT activity can be modified by both heparin and citrullination.

PN‐1, in common with PAI‐1 and A2AP, has the capacity to inhibit both thrombin and the plasminogen activators (tPA and uPA), displaying anticoagulant and antifibrinolytic properties. This specific serpin is predicted to have differing inhibitory functions dependent upon the level of activation of a target protease with honing to sites of protease activation (Aymonnier *et al*, 2021). Unlike PAI‐1 or A2AP, PN‐1 is not detected free in blood but is expressed by many cells including blood and vascular cells, notably through its binding to heparan sulfate which increases its ability to inhibit thrombin. PN‐1 is expressed in the heart and vasculature with associations to thoracic aneurysm, atherosclerosis, cardiac remodeling, heart failure, and fibrosis.

The potential of serpins for the treatment of hemophilia has been considered. Of great interest the inhibition of endogenous anticoagulant proteins, serpins such as AT or PN‐1 have emerged as an alternative approach to treating hemophilia, thus restoring the balance between coagulant and anticoagulant systems. Indeed, inhibiting endogenous anticoagulant proteins has emerged as an alternative way to treat hemophilia. A synthetic siRNA (named fitusiran) reduces circulating AT levels and has been tested in clinical trials for treatment of hemophilia. The updates, presented on the recent annual congress of the European Association for Haemophilia and Allied Disorders (EAHAD), showed positive results in a Phase 3 clinical trial. In another approach, the RCL of a human variant of A1AT called A1AT Pittsburg has been mutated to a KRK sequence converting it into a specific inhibitor of activated protein C termed SerpinPC. Results in a Phase 2 clinical trial presented at the recent EAHAD congress showed that SerpinPC was well‐tolerated and reduced bleeding in persons with severe hemophilia. Moreover, preclinical studies have suggested that inactivation of PN‐1, which is released from platelets upon activation, might also represent a novel therapeutic approach for the treatment of hemophilia by increasing thrombin activity as well as thrombin generation, directly at the site of vascular injury (Aymonnier *et al*, 2021; Maas & de Maat, 2021).

Of notable significance, the serpins expressed by viruses have been demonstrated to display a broad range of inhibitory targets and are under study as new therapeutic approaches. New serpins have in fact been identified in many organisms: in plants, insects, and bacteria as well as in the gastrointestinal tract and skin in mammals. In barley, serpins improve the foam in beer. Serpins associated with the gut microbiota are detected in ulcerative colitis. Serpins derived from other forms of life provide new insights into developing new treatments. Viruses have evolved highly efficient mechanisms to block host immune responses to viral infection, mirroring the mammalian regulatory hemostatic serpins (Yaron *et al*, 2020; preprint: Zhang *et al*, 2022). Serp‐1 and Serp‐2 are myxomavirus‐derived serpins (a rabbit poxvirus that does not infect humans), while CrmA is found in the Vaccinia virus, the virus used for smallpox vaccine development and as an expression vector. Serp‐1, as for PAI‐1, A2AP, PN‐1, and A1AT, targets a wide array of serine proteases (Fig 1). Serp‐1 binds thrombolytic and thrombotic proteases (tPA, uPA, plasmin, fXa, and thrombin (in the presence of heparin)) as well as complement proteases (demonstrated by immunoprecipitation and mass spectrometry, preprint: Zhang *et al*, 2022). Two other viral serpins, Serp‐2 and CrmA are cross‐class inhibitors of serine and cysteine proteases and inhibit apoptotic and inflammasome pathways. Serp‐1 has demonstrated efficacy in a wide range of animal models of disease (Yaron *et al*, 2020) as well as efficacy and safety in a Phase IIA clinical trial in patients who received stents for unstable coronary syndromes (Tardif *et al*, 2010). It was noted in the preclinical and clinical trials that neutralizing antibody detection was low and major adverse events were zero (MACE = 0 at the highest dose) further illustrating the targeted activity at sites of protease enzyme activation. The wild‐type Serp‐1 gene has been expressed and purified from Chinese hamster ovary (CHO) cells and has recently been modified by PEGylation to improve efficacy.

**Figure 1:**
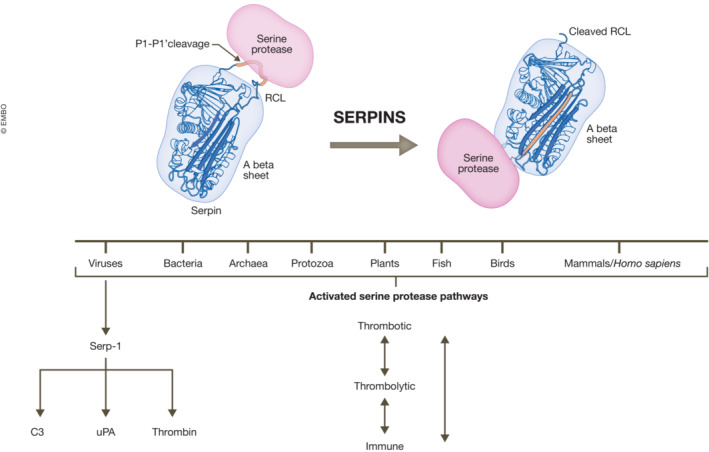
Serpins are expressed in all life forms, with many displaying broad protease inhibitory functions The host response to pathogens is achieved in part through an interplay between platelets and immune cells triggering inflammation, thrombotic and thrombolytic processes, and serine protease pathways, and many serpins have targeted these pathways. A graphic of the structure of serpins and their interactive inhibition of serine proteases is presented here to illustrate that the upper pole reactive center loop (RCL) acts as a bait for a serine protease in coagulation and complement pathways, among others. Serp‐1 is postulated to act at sites with upregulation of protease activity, to inhibit and regulate target proteases in cardiovascular, hematological, and immune pathways with the potential to restore homeostasis. uPA, urokinase‐type plasminogen activator; C3, complement 3 protease.

Serp‐1 activity is closely correlated with binding to the uPA/uPA receptor (uPA/uPAR) complex at the leading edge of macrophages providing a central role in the activation of matrix‐degrading metalloproteinases allowing immune cell invasion and releasing growth factors. Inhibition or deficiency of uPAR block Serp‐1 activity in aortic allograft transplant and wound healing models. With Serp‐1 treatments, pristane‐induced lupus lung hemorrhage and SARS‐CoV‐2 infection in mouse models are associated with reduced uPA and complement C5b‐9 membrane attack complexes on immunohistochemical analysis and altered gene expression. Serp‐2, a cross‐class serine and cysteine protease inhibitor, reduced inflammation in mouse models of arterial injury and transplant (Yaron *et al*, 2020). Serpins are inhibitors that function at sites of protease pathway activation even with low kinetic rates of association (K_ass_) demonstrating targeted inhibition at sites of protease activation. Mutagenesis of the Serp‐1‐ and RCL‐derived peptides have also demonstrated modified inhibitory activity. Viral serpins may have additional potential benefit as naturally developed platforms or designs for new therapeutics by targeting mammalian proteases that are not naturally regulated in mammals. Thus, viral serpins illustrate the capacity for broad protease targets allowing for selective and beneficial therapeutic efficacy and are posited to allow targeting at sites of protease activation. Similarly, natural biologic serpin reservoirs in viral, bacteria, and plants among others may guide new approaches for drug development.

As noted above, serpins share several unique central and cogent characteristics—(i) their role as inhibitors, acting specifically at sites where there is increased target protease activity; (ii) a unique “suicide” inhibitory mechanism of action; (iii) the ability to target a diverse array of protease targets; and (iv) the amenability of the serpin structure to allosteric fine‐tuning of inhibitory activity (Maas & de Maat, 2021).

The viral serpins have proven amazingly effective in a wide array of animal models and, as for the mammalian serpins, illustrate the unique and potentially beneficial characteristics of serpins. Few recombinant serpins, apart from the virus‐derived Serp‐1 protein, have been tested outside their natural pathways in clinical trial. In fact, the viral serpins may have arisen from recombination events between viral and mammalian genomes wherein viral genes may have been transmitted to mammals, or vice versa. Serp‐1 and Serp‐2 have demonstrated the capacity of serpins as therapeutics acting specifically at sites where there is increased target protease activity (Song & Lu, 2018; Singh *et al*, 2020; Yaron *et al*, 2020; Maas & de Maat, 2021), and as their suicide inhibitory mechanism results in the formation of a covalent complex, this obviates the risk of protease release as inhibitor concentrations decrease. This select targeting of activated proteases provides at least a partial explanation for the marked efficacy at low doses, the capacity to treat a wide array of diseases and the remarkably low adverse events.

Clearly, SERPINs as a large family are involved in many biological processes. It is generally accepted that these proteins have multiple functions in addition to the inhibition of serine proteinases. The field is now focusing on: (i) discovery of novel functions and applications of these proteins both building on known inhibitory target actions and (ii) investigations of mechanisms for the novel functions. In short, there remain many new areas for investigating and developing new therapeutic approaches in this amazingly effective and highly evolved class of regulatory proteins that incorporate the ability to target sites of protease activation in disease. The investigations will be greatly enhanced by advanced state‐of‐art technologies, including AI.

## Author contributions

**Marie‐Christine Bouton:** Conceptualization; writing – original draft; writing – review and editing. **Margarethe Geiger:** Conceptualization; writing – original draft; writing – review and editing. **William P Sheffield:** Conceptualization; writing – original draft; writing – review and editing. **James A Irving:** Conceptualization; writing – original draft; writing – review and editing. **David A Lomas:** Conceptualization; writing – original draft; writing – review and editing. **Sihong Song:** Conceptualization; writing – original draft. **Ritvik S Satyanarayanan:** Writing – original draft; writing – review and editing. **Liqiang Zhang:** Conceptualization; writing – review and editing. **Grant McFadden:** Writing – original draft; writing – review and editing. **Alexandra R Lucas:** Conceptualization; writing – original draft; writing – review and editing.

## Disclosure and competing interests statement

Dr. Lucas is the founding scientist for a biotech startup company, Serpass Biologics. Dr. Lucas and her laboratory receive no funding from Serpass at this time. David Lomas is an inventor on patent PCT/GB2019/051761 that describes the development of small molecules to block the polymerisation of Z α1‐antitrypsin.
